# Genetic signatures of a range expansion in natura: when clones play leapfrog

**DOI:** 10.1002/ece3.2392

**Published:** 2016-08-26

**Authors:** Ronan Becheler, Constance Xhaard, Etienne K. Klein, Katherine J. Hayden, Pascal Frey, Stéphane De Mita, Fabien Halkett

**Affiliations:** ^1^ UMR IAMINRAUniversité de Lorraine54000NancyFrance; ^2^ UR Biostatistique et Processus SpatiauxINRA84914AvignonFrance; ^3^ Present address: INSERM U1018, CESP, Univ. Paris‐SudUVSQ, Université Paris‐SaclayInstitut Gustave RoussyVillejuifFrance; ^4^ Present address: Royal Botanic Garden Edinburgh20a Inverleith RowEdinburghEH3 5LRUK

**Keywords:** Biological invasions, colonization, dispersal, fungal plant pathogen, genetic diversity, range expansion

## Abstract

The genetic consequences of range expansions have generally been investigated at wide geographical and temporal scales, long after the colonization event. A unique ecological system enabled us to both monitor the colonization dynamics and decipher the genetic footprints of expansion over a very short time period. Each year an epidemic of the poplar rust (*Melampsora larici‐populina*) expands clonally and linearly along the Durance River, in the Alps. The colonization dynamics observed in 2004 showed two phases with different genetic outcomes. Upstream, fast colonization maintained high genetic diversity. Downstream, the colonization wave progressively faltered, diversity eroded, and differentiation increased, as expected under recurrent founder events. In line with the high dispersal abilities of rust pathogens, we provide evidence for leapfrog dispersal of clones. Our results thus emphasize the importance of colonization dynamics in shaping spatial genetic structure in the face of high gene flow.

## Introduction

In population genetics, dispersal has long been viewed as a force that homogenizes allelic frequencies across populations and balances the effect of drift in equilibrium models (Slatkin 1985; Rousset 2004; Petit 2011). Nevertheless, colonization can also result in strong and persistent genetic structure (Ibrahim et al. 1996; Austerlitz et al. 1997; Le Corre and Kremer 1998). This exemplifies the dual role of dispersal: on the one hand colonists (Le Corre and Kremer 1998), which form a subset of the source population, and create genetic differentiation through a spatial analogue of drift (Slatkin and Excoffier 2012), and on the other hand migrants, which erode genetic differentiation among established populations, through regular gene exchanges.

The hallmark of range expansion under restricted gene flow is an erosion of genetic diversity together with an increase in genetic differentiation along axes of expansion. A particular genetic pattern during expansion is the increase in frequency of a rare variant at the front of a colonization wave, the so‐called surfing phenomenon (Klopfstein 2006; Hallatschek et al. 2007; Excoffier and Ray 2008). Surfing promotes genetic revolution during dispersal and can lead to new allele fixation even in the case of slightly deleterious mutations (Travis et al. 2007; Excoffier and Ray 2008). However, surfing is a rare phenomenon which is more likely to occur in one‐dimension expansion and assuming thin tailed dispersal (like Gaussian and Exponential kernels) (Klopfstein 2006; Fayard et al. 2009). This may be the reason why surfing has only been clearly observed in controlled conditions (Hallatschek et al. 2007). More generally, various spatial genetic patterns after range expansion are shaped by the interplay of demography (local population growth) and migration regime, especially the frequency of long‐distance dispersal (LDD) events (Bialozyt et al. 2006; Fayard et al. 2009; Hallatschek and Fisher 2014).

Most – if not all – species have experienced range expansion at some point of their history (Petit 2011). The resulting strong spatial genetic structure was observed in many taxa, including modern humans following their expansion from east Africa (Edmonds et al. 2004). However, most of these observations concerned large spatial and temporal scales and are thus decoupled from a fine‐grained description of the invasion dynamics at the origin of the spatial genetic structure. Biological invasions provide the opportunity to simultaneously examine the demographic and genetic outcomes of range expansion (Biek et al. 2007).

**Figure 1:**
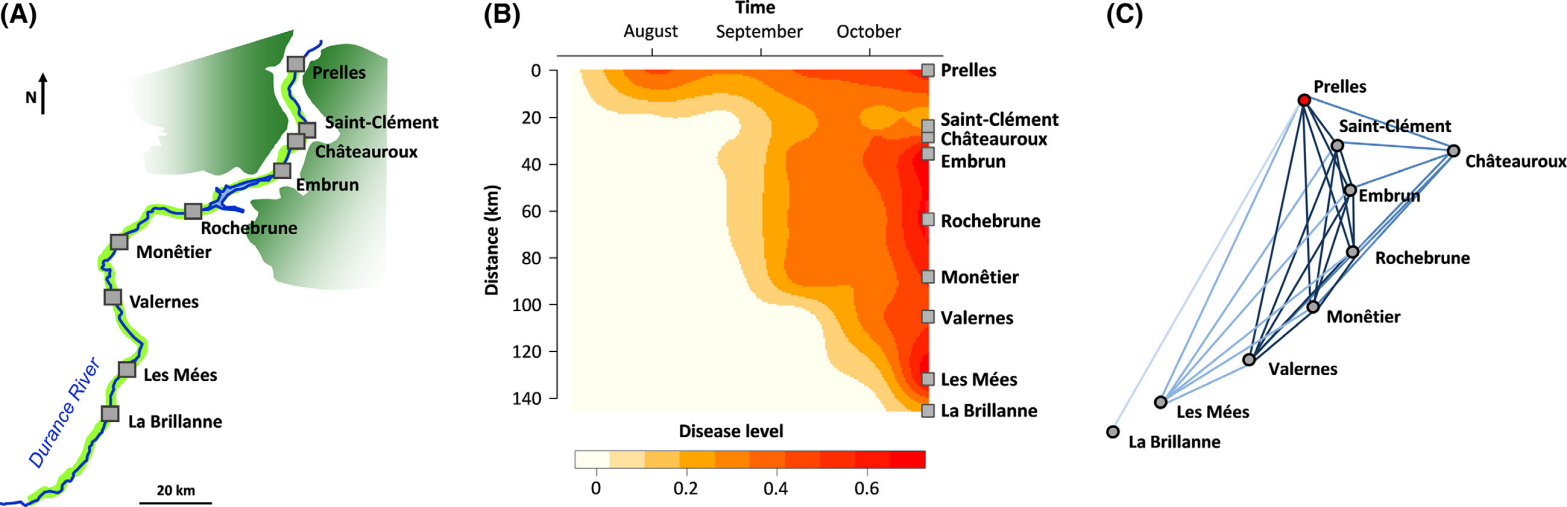
Overview of the colonization of the Durance valley by the poplar rust pathogen. (A) The Durance ecological system. The rust fungus has one round of sexual reproduction on larch trees each spring. This is followed by the annual epidemic resulting from several rounds of clonal multiplication on poplar and spread along the corridor (dark green area, larch distribution; pale green line, riparian stands of wild poplars). (B) Disease dynamics (in nine locations monitored in 2004 from early July to mid‐October). Higher incidences are represented by darker colors. (C) Network of spatial genetic structure among locations at the percolation threshold (*D*_p_ = 0.36). Nodes represent locations, whereas links represent the magnitude of genetic distance between two locations (dark blue pairwise *F*_ST_ in [0–0.1]; normal blue *F*_ST_ in [0.1–0.21]; pale blue *F*_ST_ in [0.21–0.3]; very pale blue *F*_ST_ in [0.3–0.36]).

Here, we aim to document how genetic differentiation arises in a natural population of the poplar rust fungus (*Melampsora larici‐populina*) subjected to a linear and continuous range expansion, surveyed in real time. We take advantage of a particular ecological corridor, the Durance River (southern France). This simple ecological system provides the opportunity to analyze in parallel colonization dynamics and the resulting population structure. Deeply embanked in the French Alps, the Durance River valley (ca. 145 km long and a few km wide) channels the dispersal of *M. larici‐populina* in a downstream direction (Fig. 1A). This pathogen initiates the epidemics with an annual episode of sexual reproduction on larches (*Larix decidua*), which is restricted to the uppermost part of the valley. Then, the fungus switches to asexual reproduction on poplars and performs several rounds of clonal multiplication until leaf‐fall. The early stages of the epidemic thus occur each year in the uppermost part of the valley where larches and poplars coexist, and then, the disease spreads southward, downstream in the valley. Dispersal occurs along a 1D and continuous domain, as wild poplar stands are distributed all along the riversides.

This study is the following of a previous population genetic analysis which was formerly conducted to identify population sources and keep track of pathogens' origin during poplar rust dispersal along the Durance River (Xhaard et al. 2012). Two genetically distinct groups were detected, the so‐called wild and cultivated types, originating from distinct evolutionary histories. This former work also highlighted the increasing mixing of these two types during the invasive phase, and an absence of hybridization. As these two types of individuals bear very different genetic profiles, each individual sampled along the Durance River valley can be confidently assigned a population of origin (Xhaard et al. 2012).

Here, we aimed at describing further the genetic footprint of this particular 1D‐expansion. It is noteworthy that wild poplar genotypes do not exert strong and selective selection pressure on *M. larici‐populina* populations (Xhaard et al. 2011). The evolution of allelic and genotype frequencies is mostly driven by neutral processes. This part of the analysis was lacking in Xhaard et al. (2012), as we interested primarily in tracing back the origin of migrant individuals. This study is dedicated to the reverse side of the coin, the genetic differentiation that can emerge during colonization. To this aim, we focus on the wild type only, that is, the individuals migrating strictly along the Durance River valley, and take the invasion dynamics into account. As colonization is achieved through the dispersal of clonal propagules, we will pay particular attention to the variation in clonal composition of the sampled populations. In the following, we will successively: (1) confirm that the upstream sample is indeed the source point of the epidemic through an original network approach; (2) examine in detail the variation of classical population genetic indices along the Durance River valley and parallel those profiles with the observed colonization dynamics; and (3) search for poplar rust genotypes surfing downstream the Durance River valley.

## Material and Methods

### Epidemic survey

We monitored the 2004 epidemics at nine sites along the Durance corridor. Adjacent sites were 5–28 km apart. Each site was surveyed for rust disease every 3 weeks from early July to mid‐October (six dates of disease monitoring). Disease incidence was evaluated as the number of infected leaves found over the search time elapsed during each survey.

To represent disease progression in continuous time and space, data interpolation was performed using the mba.surf function in the R‐package MBA (R Development Core Team 2014).

### Genetic analyses

#### Population genetic material

All nine populations were sampled at mid‐October to examine further their genetic characteristics at the end of the epidemic. Individuals were randomly sampled in each site and collected on different trees or distinct branches to avoid resampling clonal replicates (Barrès et al. 2012). This study is a reanalysis of a subset of previously published data (Xhaard et al. 2012) (Dryad repository; doi: 10.5061/dryad.249kj74s). We focus on the 252 indivi‐duals assigned as wild isolates in Xhaard et al. (2012). Individuals were typed for 26 microsatellite markers. Due to the very low disease prevalence at some locations, sample size was restricted to 7, 16, and 21 individuals in Saint‐Clément, Châteauroux, and La Brillanne, respectively. Sample size was nearly 30 individuals and above at other locations.

#### Clonal discrimination

As the poplar rust fungus reproduces clonally during the epidemic phase, the first step of genotypic data analysis consisted in the discrimination of clonal lineages. The simplistic screening of identical multilocus genotypes (MLGs) is not sufficient to discriminate clones, due to the very likely occurrence of scoring errors. This leads to the occurrence of distinct MLGs differing at few alleles. We thus used an approach recommended by Arnaud‐Haond et al. (2007) based on the distribution frequency of the number of distinct alleles. Using genclone 2.0 (Arnaud‐Haond and Belkhir 2006), we generated a genetic distance matrix for all pairs of MLGs within our data set and the frequency distribution of the pairwise differences in number of alleles between MLGs (Fig. S1). This distribution is bimodal, the main mode being located at 23 distinct alleles. The first small peak (between 0 and 4 distinct alleles) corresponds to pairs of MLGs which differ only slightly. These MLGs were merged into a single clonal lineage.

We further used MLgsim software (version 2.0 http://www.rug.nl/research/theoretical-biology/downloads) (Stenberg et al. 2003) to assess whether identical MLGs can occur by chance given the allelic frequency in the population (Halkett et al. 2005). No repeated MLG can be simulated after 10^9^ iterations, indicating a very high discrimination power of the marker set. Moreover, the *P*_sex_ values (probability of observing *n* times a given MLG) were very small (<10^−15^). Based on these results, we can confidently assess that all the MLGs we identified can indeed be considered as true clonal lineages.

#### Population genetic indices

For each location, we computed two complementary genotypic indices: (1) The clonal richness was estimated by *R *= (*G* − 1)/(*N* − 1), *G* being the number of clonal lineages, and *N* the number of sampled individuals; (2) the slope *β* of the Pareto distribution, summarizing the distribution of clonal membership within a population. *β* is an index of clonal evenness, describing the relative abundance among clonal lineages (Arnaud‐Haond et al. 2007). The value of this index decreases when the dominance of clonal lineages increases.

Two series of genetic analyses were performed: (1) including clonal replicates, to detect the genetic consequences of clonality; and (2) retaining a single copy of each clonal lineage. For each set, gene diversity within locations was estimated through the mean number of alleles per locus (*Â*), standardized to the smallest sample size (i.e., 7), and unbiased gene diversity (*H*_E_) (Nei 1978). Genetic structure among locations was estimated with *θ* (Weir and Cockerham 1984).

#### Population network

A location‐centered network was built with all individuals (i.e., including clonal replicates) using the *F*_ST_‐based Reynolds distance (Reynolds et al. 1983) noted *D* and calculated as follows:

$$D=-\ln(1-\theta_{w}),$$

with *θ*_w_ the weighted estimate of the pairwise coancestry coefficient, assessed as:

$$\theta_{w}=\frac{\sum_{i=1}^{k}a_{i}}{\sum_{i=1}^{k}a_{i}+b_{i}},$$

where *a*_*i*_ and *b*_*i*_ are the estimates of variances of interest between populations and within populations for the locus *i*.

The network was analyzed at the percolation threshold, automatically calculated by the software EDEnetworks (Kivelä et al. 2015). Above the percolation threshold, the network is “over‐connected” with most pairs of nodes being linked. Below the threshold, the network is split into small, disconnected clusters of populations.

## Results

The disease progressed rapidly from Prelles (the first site to be infected) down to Monêtier (≈90 km from the source) until early September (Fig. 1B). Then, it spread more slowly but regularly until the most downstream site, La Brillanne, where infection began quite late (early October). In contrast with the rapid disease spread and high incidence in the upstream part of the valley, the sites of Saint‐Clément and Châteauroux were infected later and displayed low disease incidence at the end of the epidemic (mid‐October). The whole transect was colonized within 13 weeks.

**Figure 2:**
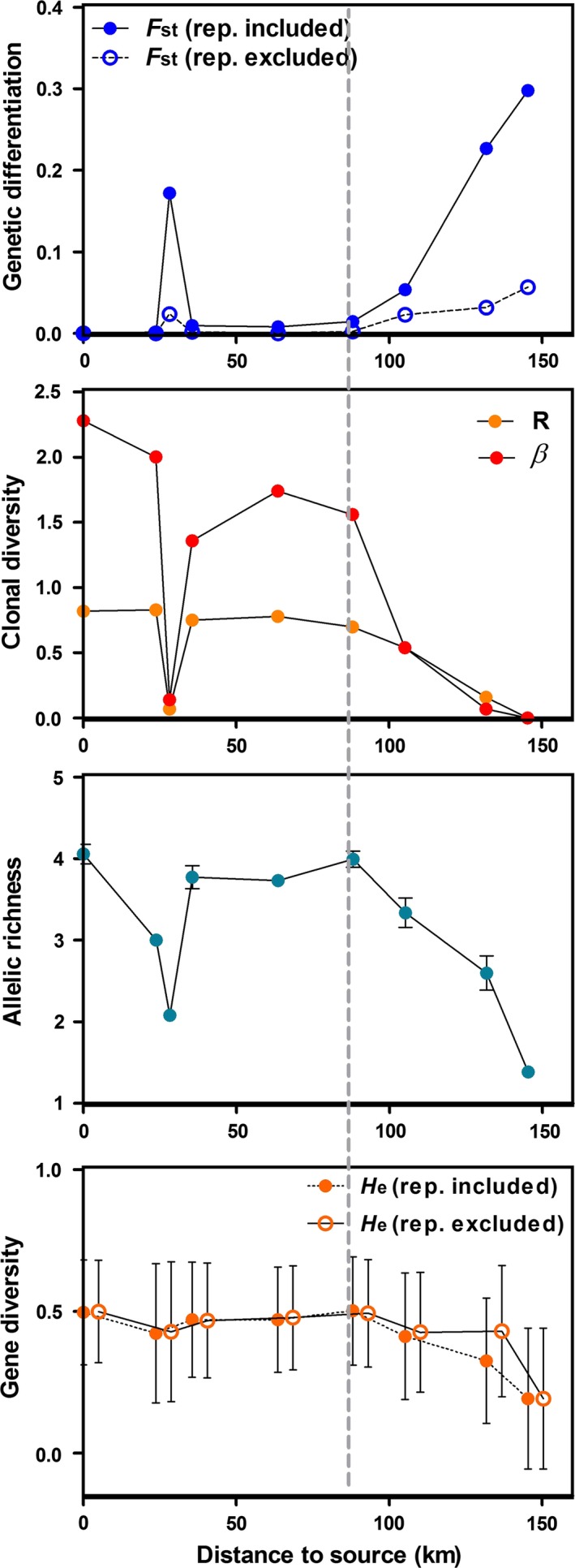
Variation in genetic indices along the Durance valley. The vertical gray dashed line indicates the so‐called break point. From top down: genetic differentiation to the upstream location (pairwise *F*_ST_ between Prelles and the downstream locations); clonal diversity (clonal richness *R* and evenness *β*); mean number of alleles per locus (*Â*), standardized to the smallest sample size (seven individuals at Saint‐Clément); gene diversity (*H*_E_). For *F*_ST_ and *H*_E_, calculi were performed including or not clonal replicates (rep. included or rep. excluded, respectively).

Genetic structure mirrors these dynamics. Prelles occupies a pivotal position in the population network (Fig. 1C), confirming its affiliation to the source region. Except for Chateauroux, which is a clear outlier, southern sites were more weakly connected. This pattern of differentiation was confirmed by the increase of pairwise *F*_ST_ downstream the valley, using Prelles as a reference (Fig. 2), reaching 0.30 for La Brillanne. Comparing the variation of all genetic indices (Fig. 2), the Durance transect clearly split into two zones, with a break point located around Monêtier. Parameter values were relatively stable upstream, but displayed consistent variation downstream from Monêtier. Châteauroux mitigated this dual pattern with discrepant values of population genetic indices.

**Figure 3:**
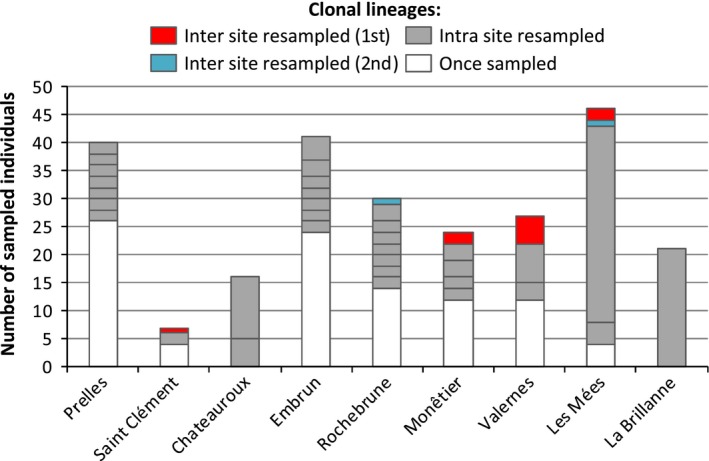
Details on the clonal composition of the sampled populations. For each location, number of unique clonal lineages, that is, found in a single copy (on the legend: “once sampled,” figured by the white boxes), number of locally abundant clonal lineages, that is, multiple clonal copies found in a single site (on the legend: “intra site resampled,” figured by the gray boxes), and sizes of the two clonal lineages found across locations (on the legend: “inter site resampled 1st and 2nd,” figured by the red and blue boxes).

We observed an increase in the number of clonal lineages sampled more than once at Châteauroux and downstream from Monêtier (Fig. 3). This erosion of clonal diversity is further illustrated by the sharp decrease in clonal richness and evenness (*R* and *β*, respectively) downstream from Monêtier (Fig. 2), which illustrates the establishment of few predominant clonal lineages. These indices rapidly reach minimal values with a single clonal lineage sampled in La Brillanne. Although those clonal lineages predominate locally, only two clonal lineages were sampled at different sites (Fig. 3), maximal resampling distance of ≈100 km), depicting a large reshuffling of clonal composition along the transect. Interestingly, the single clonal lineage in La Brillanne was never sampled upstream.

One clonal lineage (noted MLL1) was found at several instances and at distant locations (red boxes in Fig. 3), with a maximal frequency of 0.19 at Valernes. Frequencies of MLL1 were not correlated with the sampling size (*r*² = 0.14; *P *=* *0.32) suggesting that our assessment of clonal distribution was not biased by differences among sites in sampling effort. The several occurrences of MLL1 enabled to test for an increase in frequency along the invaded domain, as expected under the surfing hypothesis. However, the correlation between the frequency of MLL1 and geographical distances was very weak (*r*² = 0.02; *P *=* *0.69), indicating that this clonal lineage did not surf on the Durance River. Alternatively, we observed an erratic distribution of MLL1 along the invaded domain, which may result from LDD events or from the limited sample size. We therefore tested whether the lack of detection of MLL1 between Saint‐Clément and Monêtier could be due by chance. Assuming an even distribution of this lineage along the invaded domain, the probability to detect it given the sample size follows a Binomial law [*X *~ *B*(*n*, *p*), with *X*, the occurrence of MLL1; *n*, the sample size and *p*, the frequency of MLL1]. The global frequency of MLL1 was set at 0.04 (10 occurrences over 252 samples), which amounts to the most conservative estimates. The probability that we observed by chance no individual belonging to MLL1 between Chateauroux and Rochebrune (total of *n *=* *87 sampled individuals) was very low (*P*_B(*X*=0)_ = 0.03). The gap in MLL1 detection between Saint‐Clément and Monêtier would not result from a limited sample size. The resampling of MLL1 at very distant sites would thus provide evidence for a leapfrog dispersal event.

## Discussion

Because of the unique nature of the Durance River system, we were able to observe the entirety of a range expansion and to grasp the origin of genetic differentiation from the source population.

The rust invasion was characterized by a two‐phase dynamic. Upstream, fast colonization fostered gene flow and impeded the creation of a spatiotemporal structure among sampling sites. Those locations thus retained the initial level of clonal and gene diversities and, as expected under an island model of colonization with high gene flow (Le Corre and Kremer 1998), did not display genetic differentiation. The second phase was marked by the loss of impetus of the expansion wave downstream from Monêtier. Slowed colonization dynamics created an age structure among founded populations, resulting in an increase of genetic differentiation associated with an erosion of genetic diversity along the invaded domain. This illustrated – at small spatial and temporal scales – the consequences of successive founding events expected under a stepping‐stone model of colonization (Le Corre and Kremer 1998) and usually documented in phylogeographic studies of postglacial recolonizations or bio‐invasions (Leblois et al. 2000; Neiva et al. 2010; Petit 2011; Fontaine et al. 2013). The break we observed in the genetic signatures along the range expansion exemplifies the importance of colonization dynamics in shaping population genetic structure.

Theoretically, the distribution of individual invasive success is linked to effective population size (*N*_e_) (Klopfstein 2006; Fayard et al. 2009). Distributions with low variance, such as observed under frequent LDD, lead to a better conservation of genetic diversity (high *N*_e_) (Fayard et al. 2009; Berthouly‐Salazar et al. 2013; Nullmeier and Hallatschek 2013), as we observed upstream to the break point. Assuming the migration regime is fixed, the loss of genotypic diversity and the rise of spatial genetic differentiation we observed downstream and in Châteauroux would result from an increase in the variance of the reproductive success, because of more recent and pronounced founding events. Interestingly, a comparable dichotomy in the spatial domain was found in a simulation study (Bialozyt et al. 2006) for high frequencies of LDD (10^−4^ to 10^−2^).

Narrow corridors, such as the Durance Valley, are expected to raise the odds of surfing events (Fayard et al. 2009). Yet, we detected no increase in frequency of any genotype. Conversely, we observed a patchy distribution of clonal lineages that predominate locally but are reshuffled across sampling sites. Moreover, the single clonal lineage sampled at the most downstream site was not found upstream. These elements did not support the surfing hypothesis but rather point to frequent events of LDD, in line with the high dispersal abilities of rust pathogens (Brown and Hovmøller 2002). In this scenario, clonal lineages would jump over already colonized sites to found new foci downstream (Fayard et al. 2009). This is illustrated by the resampling of clonal lineages at distant sites, without a marked increase in frequency downstream the valley. During the Durance River colonization, rust clones did not surf but played leapfrog.

## Conclusion

The poplar rust colonization of the Durance River valley is a neat ecological system to decipher the population genetic outcome of range expansion. In this study, we showed how the colonization dynamics paralleled the variation in population genetic indices. Depending on the demographic scenario of colonization, some sites were at genetic equilibrium, other displayed strong hallmarks of founder event even if they were colonized early. Our main result was that the increase in genetic differentiation went hand in hand with an erosion of genotypic diversity along the invaded domain. The next step will consist in examining several instances of colonization to gain more insights on the interplay between colonization dynamics, dispersal regime, and genetic consequences of the Durance invasion. It is much likely that the strength of the epidemics and the colonization dynamics would differ among years. It would thus be very interesting to compare the resulting population genetic structure to identify the common features and differences across years. Do we always observe an increase in genetic differentiation? Is the slope of increase inversely proportional to the speed of colonization? Is the patchy distribution of clonal lineage a general rule? Is it possible that some genotype surf the valley after all?

## Conflict of Interest

None declared.

## Supporting information


**Figure S1.** Frequency distribution of the number of different alleles between pairs of MLGs of poplar rust.
