# Supplementary material for: Genetic signatures of a range expansion in natura: when clones play leapfrog
Source: Ecol Evol. 2016 Aug 26;6(18):6625–32. doi: 10.1002/ece3.2392 (PMC5058533; doi:10.1002/ece3.2392)
Supplement: Supplementary file 1 — Figure S1. Frequency distribution of the number of different alleles between pairs of MLGs of poplar rust. [file ECE3-6-6625-s001.docx]

**Supporting Information**


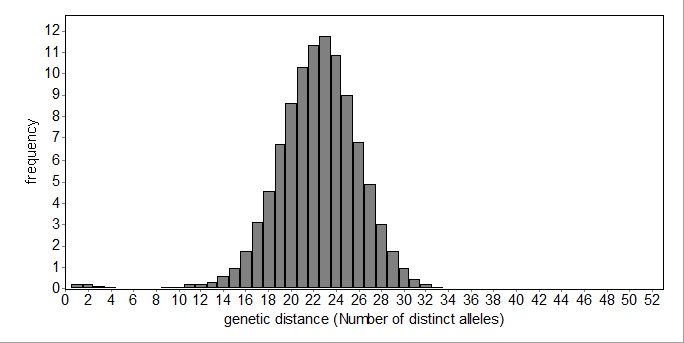


**Figure S1** Frequency distribution of the pairwise number of alleles differences between MLGs of poplar rust. This distribution is bimodal, containing a first small peak at very small genetic distances (< 4 alleles). The slightly differing MLGs, whose pairs generated this small peak, were merged together into the same clonal lineages.
